# Inner Ear Function Evaluation in Mobile Phone Users: A Cross-Sectional Study From a Tertiary Care Centre in North India

**DOI:** 10.7759/cureus.51573

**Published:** 2024-01-03

**Authors:** Nitin Sharma, Bhawana Pant, Mohammad Mohsin Raza, Avanish Chamoli

**Affiliations:** 1 Department of Otolaryngology, Head and Neck Surgery, Government Doon Medical College, Dehradun, IND

**Keywords:** electromagnetic waves, pure tone audiometry, otoacoustic emissions, inner ear function, mobile phones

## Abstract

Background

India has approximately 1.02 billion mobile phone users. The electromagnetic radiations emitted by telecommunication systems are absorbed by the recipient's body, leading to changes in brain electrical activity, sensations of warmth or burning around the ear, and alterations in the blood-brain barrier. The inner ear, being the closest organ during mobile phone use, directly receives these electromagnetic radiations. This study aims to assess the inner ear function among mobile phone users, investigate the impact of mobile phones on the hearing thresholds of volunteers through pure-tone audiometry (PTA), and delve into the same using otoacoustic emissions (OAE).

Methodology

A cross-sectional study was conducted at a single center in North India from September 2020 to March 2021. The sample size of around 100 was determined using G Power software (G Power, Aichach, Germany), including volunteers aged 18-25, using mobile phones for over a year with normal hearing. Exclusions involved various ear-related histories or chronic systemic illnesses. Dominant and non-dominant ear groups were formed based on mobile phone usage. The study involved comprehensive ENT examinations, pure-tone audiometry, and otoacoustic emissions. We performed statistical analyses using SPSS version 22.0 (IBM Corp., Armonk, NY), which presented descriptive statistics and employed tests for group comparisons.

Results

Most participants were in the 21-23 age group (56%), with a mean age of 22.16 ± 1.77 years. There were 45 males and 55 females. The mean mobile phone usage was 6.6 ± 1.98 years, with varying daily durations. The dominant ear for mobile phone usage was predominantly the right ear (75 participants). Pure-tone audiometry results showed no statistically significant differences between dominant and non-dominant ears. Among the 24 participants with absent OAE, no significant association was found with mobile phone usage duration. Notably, the highest incidence of absent OAE occurred in the 120-180-minute usage category.

Conclusion

Mobile phones have seamlessly integrated into the lives of individuals, witnessing an exponential increase in users over time. The inner ear, situated in proximity to mobile phone usage, is of particular concern. While there is existing evidence indicating potential adverse effects of mobile phones on the inner ear, further long-term studies involving larger populations are essential to comprehensively evaluating the impact on inner ear function among mobile phone users.

## Introduction

Mobile phones have become an integral part of everyone’s life. In India, there are around 1.02 billion mobile phone users as of 2016 [1]. The growing accessibility of affordable smartphones has led to an upsurge in their usage, expanding beyond mere communication to encompass internet services, gaming, the global positioning system (GPS), and various productivity and recreational activities. While these devices have undoubtedly enhanced convenience and productivity, their prolonged usage has also raised concerns regarding a cascade of potential health hazards [1].

Mobile phones use electromagnetic signals to receive and transmit signals in various radiofrequency bands. Although the electromagnetic waves are within an acceptable range, the data on the long-term effect is still lacking [2]. The operating frequency of mobile phones ranges from 500 to 2700 MHz, with peak powers between 0.1 and 2 watts [3]. The electromagnetic radiation emitted from the telecommunication system is absorbed by the recipient body and also brings about changes in brain electric activity and produces local effects on the skin and ear [1,3-5]. Utilizing hands-free options maintains a distance of 30-40 cm between the mobile phone and the user, resulting in lower exposure to radiofrequency fields compared to holding the phone close to the ears [5]. The Specific Absorption Rate (SAR) serves as the metric for measuring the radiofrequency dose received by an individual, expressed in power (Watts) per unit of tissue mass (kilograms). The SAR tolerance limit has been set to a limit of 10 W/kg by radiological protection committees from various countries. One can check one’s mobile’s SAR by typing *#07#. [3] A person using a mobile phone away from the body will have lower exposure to radiofrequency waves than someone holding the mobile phone closer [3].

Recent studies have raised concerns regarding the adverse effects of mobile phone usage, including the possible link to an increased risk of vehicular accidents, leukemia, sleep disturbances, and more serious brain tumors [4]. The inner ear is closest to mobile phone use and is a direct recipient of electromagnetic radiation. The hair cells in the Organ of Corti do not have regenerative potential, so the changes produced can be irreparable [2]. There are limited studies available evaluating the impact of continuous mobile phone usage on inner ear and hair cell function. With this study, the author aims to study the impact of mobile phones on the hearing thresholds of the volunteers using pure-tone audiometry (PTA) and explore the same using OAE.

## Materials and methods

A single-center-based cross-sectional study was conducted in North India between September 2020 and March 2021. The sample size was determined using G Power Statistical Analysis software, keeping an effect size of 0.5, an alpha error of probability of 0.10, and power to be 0.90, leading to a rounded-off sample size of around 100. The participants were selected by the convenience sampling method.

The volunteers included in the study were of either sex and were between the ages of 18 and 25. They used mobile phones for more than a year with apparently normal hearing in both ears. The volunteers with a history of chronic ear disease, a history of any ear disease or deafness, any history of ear surgery, a history of prolonged exposure to loud noise, a history of use of any ototoxic medications, any history of a chronic systemic illness known to cause hearing loss or a recent history of an ear, nose, or throat infection were excluded from the study. As it was difficult to find nonmobile users, we compared the findings of the dominant ear with those of the non-dominant ear, depending on the history of mobile phone usage. While using the mobile phone, electromagnetic absorption is maximum on the side where the mobile phone is held, and absorption decreases to 1/10th on the opposite side [2]. The ear that was frequently used was considered the dominant ear as it received the most radiation.

All volunteers underwent a closed questionnaire regarding their use of mobile phones. All volunteers underwent a complete ENT examination, including an otoscopic examination followed by PTA at frequencies of 250, 500, 1000, 2000, 4000, and 8000 Hz, and an otoacoustic emissions (OAE) study.

Following this, the volunteers were categorized into two groups: the dominant group, encompassing hearing results of the dominant ear, including those without any side preference, and the non-dominant group, comprising hearing results of the non-dominant ear. Side preference was determined based on which ear was more frequently used during phone calls. The audiologist was blinded, unaware of the dominant ear. Subsequently, the hearing results of the dominant ear were compared with those of the non-dominant ear for analysis.

The study was approved by the Institutional Ethics Committee (IHEC) of GDMC with IEC no. GDMC/2020/92 and the subjects were recruited after detailed informed consent.

Statistical analyses were conducted employing SPSS Version 22.0 (IBM Corp., Armonk, NY). Descriptive statistics, including the number of patients and percentages, were presented for discrete variables. The normality of the distribution was assessed using the Shapiro-Wilk test. Additionally, the paired Student t-test and chi-square test were employed to compare the variables among the two groups.

## Results

Demographic characteristics

**Figure 1:**
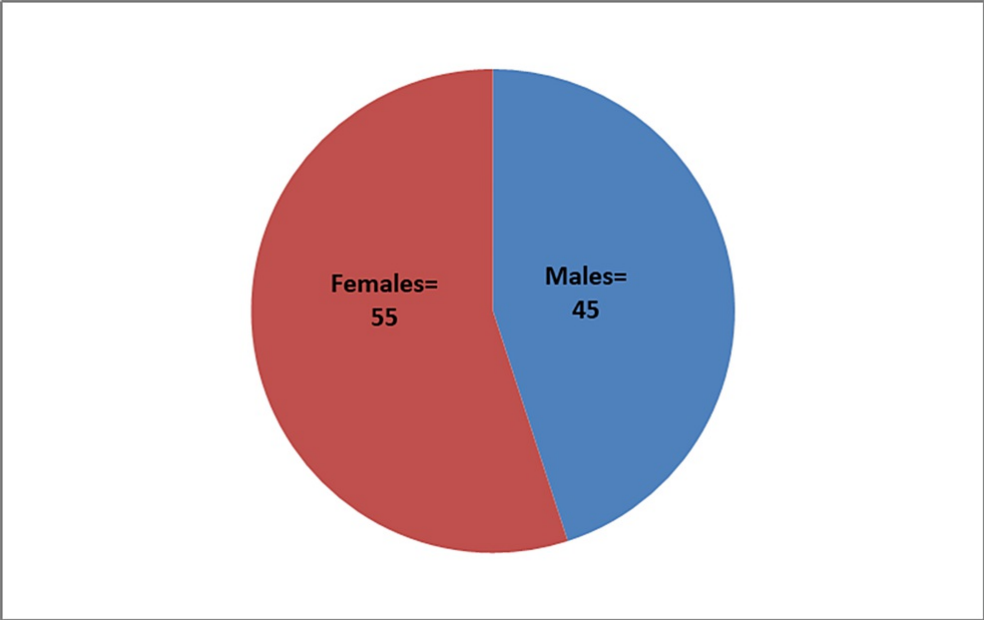
Sex distribution of participants for the study

The age distribution of participants in the study, as presented in Table 1, reveals a concentration of volunteers between the ages of 21 and 23, constituting 56% of the total. The mean age of the participants was 22.16 ± 1.77 years. The gender distribution, depicted in Figure 1, demonstrates a balanced representation, with 45 males and 55 females out of 100 volunteers, maintaining a ratio close to 1:1.22.

**Table 1:** Age distribution of participants for the study

Age Group	Males	Females	Total	Percentage (%)
18–20 years	6	12	18	18%
21–23 years	25	31	56	56%
24–25 years	14	12	26	26%
Total	45	55	100	100%

Mobile phone usage and ear preference

**Figure 2:**
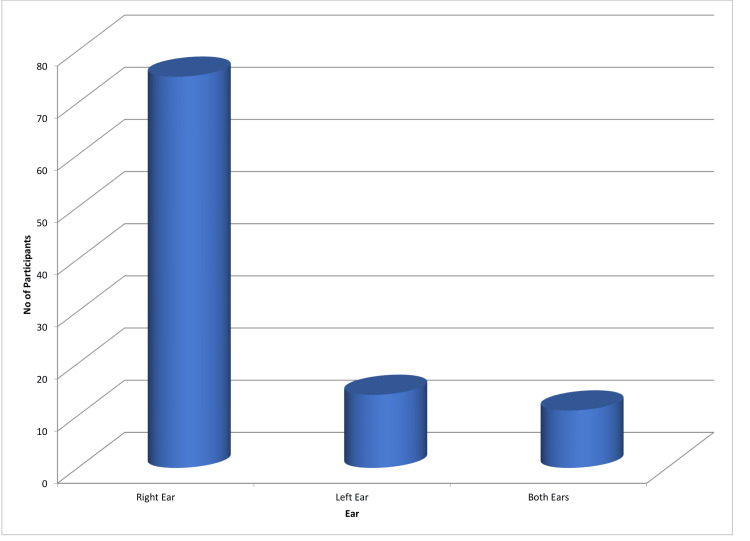
Dominant ear for mobile phone usage

Table 2 outlines the mean usage duration of mobile phones, indicating an average of 6.6 ± 1.98 years, with some participants reporting usage durations of up to 180 minutes per day. Figure 2 highlights the dominant ear for mobile phone usage, with the right ear being dominant in 75 participants, the left ear in 14 participants, and 11 participants exhibiting no preference for either side.

**Table 2:** Duration of usage of mobile phones

Usage (min/day)	No. of participants
Less than 60 mins	16
60–120 mins	41
120–180 mins	43

Audiometric measurements

For audiometric measurements, the bone conductance threshold in the dominant ear group (including those with no ear preference) was 7 ± 2.45 dB, and in the non-dominant group, it was 7.05 ± 2.75 dB. The air conduction threshold for the dominant ear group was 11.26 ± 2.58 dB, and for the non-dominant ear group, it was 12.13 ± 2.26 dB. The air-bone gap for the dominant ear group was 4.39 ± 2.29 dB, and for the non-dominant ear group, it was 4.98 ± 2.5 dB, with a non-significant p-value of 0.2382, as determined by the Student’s paired t-test (Table 3).

**Table 3:** Hearing threshold of the participants on pure-tone audiometry

Hearing threshold level	Dominant group	Non-dominant group
Bone conduction	7 ± 2.45 dB	7.05 ± 2.75 dB
Air conduction	11.26 ± 2.58 dB	12.13 ± 2.26 dB
Air bone gap	4.39 ± 2.29 dB	4.98 ± 2.5 dB

In this study, we observed the absence of OAE in a cohort of 24 participants. Among these participants, 11 individuals (11%) exhibited absent OAE in the dominant ear group, while 5 participants showed absent OAE bilaterally, with no discernible preference for mobile phone usage. Additionally, 8 participants demonstrated absent OAE in the non-dominant ear, resulting in a non-statistically significant p-value of 0.1729 as determined by the Chi-square test.

When examining the association between the duration of mobile phone usage and the absence of OAE, we obtained a non-statistically significant p-value of 0.410656 through the Chi-square test. Notably, the highest incidence of absent OAE was observed among individuals who used mobile phones for durations ranging from 120 to 180 minutes, as illustrated in Table 4. Overall, our findings suggest a potential association between mobile phone usage and absent OAE, particularly in specific duration ranges.

**Table 4:** Association of duration of usage of mobile phones with OAE

Usage (min/day)	No. of participants	OAE absent
Less than 60 mins	16	2
60–120 mins	41	7
120–180 mins	43	15
Total	100	24

## Discussion

The prevalence of mobile phone usage is steadily increasing, with these devices serving as miniature computers in everyone's pocket, facilitating voice calls, text messages, and internet services [6]. However, concerns have arisen regarding exposure to radiofrequency electromagnetic fields (RF-EMF) during mobile phone use, leading to a spectrum of symptoms such as headaches, nausea, dizziness, and fatigue [6]. Notably, concerns have arisen regarding the potential impact of electromagnetic field (EMF) exposure, linking it to sleep disturbances, vehicular accidents, and brain tumors, particularly acoustic neuromas, due to the close proximity of the acoustic nerve to mobile phones [4]. Studies suggest that the ear closest to the electromagnetic radiation source is more susceptible, with absorption significantly decreasing on the opposite side of the head [2]. While it is common practice to keep mobile phones at a distance of 30-40 cm from the body during activities such as text messaging or internet use, which effectively reduces exposure to radiofrequency fields in comparison to holding them against the ear, the potential risk of adverse effects persists [3].

In this study, the majority of participants, mainly aged 21-23 (56%), exhibited a mean age of 22.16 ± 1.77 years, with a gender distribution of 45 males and 55 females. The average duration of mobile phone usage was 6.6 ± 1.98 years, with a range of daily usage durations. Interestingly, the right ear was predominantly favored for mobile phone usage among 75 participants. Research on the long-term impact of mobile phone usage on hearing function is limited. The study by Al-Khlaiwi et al. reported prevalent symptoms like headache, sleep disturbance, tension, fatigue, and dizziness. Interestingly, our study found no evidence of symptoms related to prolonged mobile phone usage among participants [7].

Pure-tone audiometry results revealed no statistically significant differences between dominant and non-dominant ears. Despite variations in daily usage, 24 participants displayed absent OAE, and intriguingly, no significant association was established between OAE absence and mobile phone usage duration. Notably, the 120-180 minute usage category demonstrated the highest incidence of absent OAE. Hearing function assessment through pure-tone audiometry has revealed abnormal thresholds at higher frequencies among similar participants (above 8 to 16 kHz) [2]. Studies by Jadia et al. [3], Hegde et al. [8], Callejo et al. [9], and others highlighted sensorineural hearing loss in mobile phone users compared to non-users. Our research discerned variations in bone conduction thresholds, air conduction thresholds, and air-bone gaps, revealing noteworthy distinctions between dominant and non-dominant ears, albeit without reaching statistical significance. As an illustrative instance, in the context of OAE, eight participants manifested the absence of OAE in the non-dominant ear, resulting in a non-statistically significant p-value of 0.1729, as determined by the Chi-square test. Similarly, the examination of the relationship between the duration of mobile phone usage and the absence of OAE yielded a non-statistically significant p-value of 0.410656, determined through the Chi-square test [3,8-11]. On similar lines, Ramya et al. [10] also found that there was a significant increase in the hearing thresholds at all frequencies for air conduction and bone conduction in the right ear (test group) compared with the control group. Exploring OAEs as a reliable measure of cochlear function, research by Panda et al. [12]. Alsanosi et al. [13] have indicated associations between mobile phone usage and the absence of OAE. Notably, our study aligns with and supports these findings [11-13]. Additionally, studies on auditory brainstem responses revealed prolonged latency in individuals using mobile phones for extended periods, while short-term exposure seemed to have no significant impact [4,14-19]. Our findings further align with Kerekhanjanarong et al. [18], who found that subjects who used a mobile phone for more than 60 minutes per day had a worse hearing threshold for the dominant ears than the non-dominant ears, showing a direct relationship with time of exposure. Our participants also showed a similar association.

As mobile phones continue to be an integral part of individuals' lives with a surging user base, this study emphasizes the need for concern regarding the inner ear, given its proximity to mobile phone usage. Although existing evidence hints at potential adverse effects on the inner ear, comprehensive, long-term studies involving larger populations are imperative to fully understand and evaluate the impact of mobile phone usage on inner ear function.

In summary, our study, although recognizing limitations such as a small sample size and the absence of a non-mobile phone user control group, adds to the growing body of evidence indicating the potential impacts of mobile phone usage on hearing function. The identified variations in audiometric parameters and the absence of OAE highlight the need for continued research and vigilance regarding the widespread use of mobile phones. Further investigation is essential to better understand the implications of mobile phone usage on hearing health.

## Conclusions

This study provides valuable insights into the potential adverse effects of mobile phone usage on inner ear function. The findings indicate a proportional relationship between the duration of mobile phone usage and its impact on the dominant ear. Conducted on a cohort of healthy young adults, the study highlights a noteworthy observation: even mild losses in inner ear function at a younger age pose a heightened risk of future hearing impairment. Considering the escalating reliance of the younger population on mobile phones, it becomes imperative to adopt safer usage practices. Encouraging the use of hands-free devices during calls, imposing limitations on daily phone usage, and implementing restrictions on mobile phone usage for children emerge as crucial measures. The study suggests that mobile phones could potentially act as a risk factor for early onset hearing loss, possibly attributed to electromagnetic radiation.

To mitigate potential risks, it is essential to advocate for responsible mobile phone usage. While our study sheds light on these concerns, we acknowledge the need for more comprehensive, long-term studies involving larger and more diverse populations, including control groups not using mobile phones. Such investigations will provide a more thorough understanding of the adverse effects of mobile phones on inner ear function, helping to formulate informed guidelines for safer technology usage.
